# Caregiver Recognition of Childhood Diarrhea, Care Seeking Behaviors and Home Treatment Practices in Rural Burkina Faso: A Cross-Sectional Survey

**DOI:** 10.1371/journal.pone.0033273

**Published:** 2012-03-13

**Authors:** Shelby E. Wilson, Césaire T. Ouédraogo, Lea Prince, Amadou Ouédraogo, Sonja Y. Hess, Noël Rouamba, Jean Bosco Ouédraogo, Stephen A. Vosti, Kenneth H. Brown

**Affiliations:** 1 Department of Nutrition, University of California Davis, Davis, California, United States of America; 2 Institut de Recherche en Sciences de la Santé, Bobo-Dioulasso, Burkina Faso; 3 Department of Agricultural and Resource Economics, University of California Davis, Davis, California, United States of America; 4 Helen Keller International, Dakar, Senegal; Aga Khan University, Pakistan

## Abstract

**Introduction:**

To design effective national diarrhea control programs, including oral rehydration solution (ORS) and therapeutic zinc supplementation, information is needed on local perceptions of illness, external care seeking behaviors, and home treatment practices.

**Methods:**

A cross-sectional, community-based household survey was conducted in the Orodara Health District, Burkina Faso. Caregivers of 10,490 children <27 months were interviewed to assess child diarrhea prevalence and related care practices. Characteristics of households, caregivers, children, and reported illnesses were compared for those caregivers who did or did not recognize the presence of diarrhea, as defined according to clinical criteria (≥3 liquid or semi-liquid stools/day). Multiple logistic regression models were used to examine factors associated with illness recognition and treatment.

**Results:**

Clinically defined diarrhea was present in 7.6% (95% CI: 7.1–8.1%) of children during the 24 hours preceding the survey but recognized by only 55% of caregivers. Over half (55%) of the caregivers of 1,067 children with a clinically defined diarrhea episode in the past 14 days sought care outside the home; 78% of those seeking care attended a public sector clinic. Care was sought and treatment provided more frequently for children with fever, vomiting, anorexia, longer illness duration, and those living closer to the health center; and care was sought more frequently for male children. 80% of children with recent diarrhea received some form of treatment; only 24% received ORS, whereas 14% received antibiotics. Zinc was not yet available in the study area.

**Conclusions:**

Caregivers frequently fail to recognize children's diarrhea, especially among younger infants and when illness signs are less severe. Treatment practices do not correspond with international recommendations in most cases, even when caregivers consult with formal health services. Child caregivers need additional assistance to recognize diarrhea correctly, and both caregivers and health care providers need updated training on current diarrhea treatment recommendations.

## Introduction

Diarrhea is the second leading cause of death among children less than 5 years of age in lower income countries, resulting in ∼1.3 million deaths globally each year, mostly in Africa and South Asia. [1], [2] The majority of these deaths are due to fluid loss and secondary dehydration, and could be prevented with early diagnosis and timely administration of oral rehydration therapy, zinc supplementation, continued feeding of an appropriate diet, and appropriate antimicrobial therapy for dysentery, as recommended by the World Health Organization (WHO) and UNICEF. [3], [4] Children less than 5 years of age in sub-Saharan Africa experience a median of five episodes of diarrhea per year, with the highest incidence occurring in children aged 6–23 months. [5] In addition to the direct health burden of these illnesses, longer-term effects of diarrhea include secondary malnutrition [6], [7] and impaired cognitive development. [8]

Beginning in the 1990s, WHO and UNICEF initiated a strategy known as the Integrated Management of Childhood Illness (IMCI) to improve the quality of care provided in health facilities for the five conditions responsible for 70% of child deaths, namely, pneumonia, diarrhea, malaria, measles, and malnutrition. [9], [10], [11] Because the coverage of existing health facilities is still insufficient to manage all children suffering from these conditions, the IMCI program has been expanded to focus on improving family and community practices related to child health, nutrition, and development. [9], [12], [13] Early and accurate recognition of illness and timely administration of appropriate treatment, including increased fluids and continued feeding, by caregivers are critical elements to preventing child deaths. [14] To encourage greater involvement of families in diarrhea management, diarrhea control programs require information on the factors that influence caregivers' recognition and treatment of illness. [15]

The objectives of the present study were to assess caregivers' recognition and management of acute diarrhea in children residing in a rural health district of Burkina Faso, and to determine the factors related to illness recognition, care seeking behaviors and treatment practices. For the present analyses we hypothesized that: 1) caregivers currently underestimate the prevalence of diarrhea, as defined using standard clinical criteria of ≥3 liquid or semi-liquid stools per day; 2) caregiver recognition of diarrhea varies by household economic status and distance to health center, other caregiver and child characteristics (e.g., caregiver age, education, parity, ethnicity, religion, child age and sex), and the nature of the illness and its associated symptoms; and 3) case management practices (i.e., care seeking and treatment) vary by some of the same household, caregiver, child, and disease factors.

## Methods

### Study Population and Design

A cross-sectional, community-based household survey was conducted from April to September, 2010 in selected communities surrounding 24 health centers in the southern half of the Orodara Health District in southwestern Burkina Faso, with the full support of local governmental and community leaders. A two-stage procedure was used to identify households with children <27 months of age. Initially, 24 of the 25 health centers located <90 minutes (one-way) by vehicle from the District Health Office were selected based on their accessibility. In the second stage, individual communities in each health center catchment area were selected based on accessibility by vehicle to the respective health center. A total of 106 communities (73% of all communities in the selected health centers' catchment areas) were included in the survey. According to previously collected district census data (Orodara Health District, 2009), the total population of the 106 selected communities was approximately 150,000 people, representing approximately 88% of the total population in the southern half of the Orodara Health District.

The study population consisted of primary caregivers of children <27 months of age, who were identified via house-to-house visits by trained interviewers. In each community, a local guide was hired to lead the survey teams, with the aim of visiting 100% of eligible concessions. A “concession” was defined as a family compound, comprised of one or more households; and a household was defined as a group of people, related or not, living under one roof (or in the same plot), who reportedly recognized the authority of a single individual called the head of household. The survey teams re-visited households when necessary to reach participants. Only if there was no response after three attempts, the household was dropped from consideration. A total of 10,454 caregivers of all identified children <27 months of age were interviewed. Questions on case management of childhood diarrhea were restricted to the subset of 1,067 children who reportedly experienced an episode of clinically defined diarrhea (≥3 liquid or semi-liquid stools per day) in the preceding two weeks.

According to the observed point prevalence of clinically defined diarrhea in the previous 24 hours (7.6%) and the two-week period prevalence (10.2%), the available sample was sufficient to detect a difference of two percentage points in factors associated with illness recognition and treatment practices, when the factors were present in 20–80% of the population.

### Survey Instrument Design and Administration

The survey questionnaire was developed in French, and revised following pretesting. The interviews were completed in French or Dioula (the predominant local language). Twenty-seven locally hired interviewers, divided into seven teams, administered the survey. Information was collected on social and demographic characteristics of the concessions and households and the child's recent illness history.

Caregivers were questioned regarding their recollection of the number and consistency of the child's stool (*boo* and *bô*, the words for stool, and *bodji*, the term for liquid stools in Dioula, were used), the presence of blood in the stool, and the occurrence of fever, vomiting, or decreased appetite in the 24-hours period prior to the interview. For the purpose of evaluating caregiver recognition of illness, caregivers were asked later in the interview whether they thought the child had diarrhea during the previous day (*konoboli*, the term for diarrhea in Dioula, was used). The questionnaires were structured so that there was no mention of the word “diarrhea” until after the symptom-based recall. For the data analyses, diarrhea was defined in two ways: according to both the clinical definition (based on reported stool number and consistency) and the caregiver definition (based on the caregiver perception of illness), and for two time periods, namely the previous 24 hours and the past 14 days. The clinical definition was based on the criteria of ≥3 liquid or semi-liquid stools per day, as recommended by the WHO; [3] the caregiver definition was based on the response to the question about whether the caregiver thought the child had diarrhea.

Later during the interview, the caregivers were read the WHO (“clinical”) definition of diarrhea; [3] and they were then asked whether or not their child had diarrhea in the past two weeks, according to this definition. Caregivers who responded positively to this query were subsequently questioned on case management practices. Among this latter subset of caregivers, questions were posed regarding care seeking outside the home, any treatments administered (including knowledge of ORS) and child feeding practices during illness. Specifically, caregivers were asked whether the infant was breastfed and whether particular fluids or foods were given or withheld in relation to the illness.

The Institutional Review Boards of the Centre Muraz in Bobo-Dioulasso (Burkina Faso) and the University of California, Davis (USA) approved the survey as part of a subsequently initiated zinc supplementation study. Prior to administering the questionnaire, a disclosure statement was read to each of the interviewees, indicating that participation in the study was voluntary, and oral consent to participate was requested. Because the research involved minimal risk and most respondents were non-literate, the IRBs approved oral consent procedures, which were documented on each questionnaire.

### Statistical Analysis

Data were double entered and verified using EpiData Entry version 2.0 (Odense, Denmark). SAS version 9.2 (SAS Institute, Cary, NC, USA) was used for all analyses. Univariate and bivariate descriptive analyses were completed for all relevant variables. Child age was determined by the survey date minus date of birth, which was determined from the child's birth certificate or health card or through a local events calendar if the child's birth certificate or health card was unavailable (13.1% of cases).

Economic status was characterized using data on assets owned by the household (plow, cart/wagon, sprayer, donkey, cow, sheep, goat, television, solar panel, radio, portable telephone, bicycle, and motorcycle) and housing characteristics (materials used to construct walls, floors, roofs, and drinking water supply, and waste disposal facilities for the concession). [16] Factor analysis was used to assess which variables were correlated, and principal component analysis was used to construct a single summary variable representing household and concession assets and infrastructure as a proxy for relative economic status among households and concessions. This variable was then split into terciles to create a categorical variable indicating households and concessions with high, medium, or low economic status.

Sensitivity and specificity were used to examine the accuracy of caregivers' recognition of diarrhea in relation to the clinical criteria. To ascertain whether child or caregiver characteristics explained differences between caregivers who did and did not recognize the presence of clinically defined diarrhea, chi-square tests were calculated. Multiple logistic regression models then were used to estimate the probability that caregivers' perceptions of diarrhea were affected by the presence or absence of other clinical symptoms, and to examine factors associated with care seeking behaviors and treatment practices for diarrheal episodes. The probability that a caregiver recognized diarrhea as an illness was analyzed using caregiver report of diarrhea (given that the child passed ≥3 liquid or semi-liquid stools during the previous 24 hours) as the dependent variable. The probability that a caregiver sought external care for a child (given the presence of clinically defined diarrhea during the past 14 days) was analyzed using caregiver report of care seeking outside the home as the outcome, and the probability that a caregiver provided treatment (any treatment, ORS, and antibiotic) was examined using caregiver report of the particular treatment. The presence and number of clinical symptoms (blood in the stool, fever, vomiting, or decreased appetite), duration of illness, child age and sex, maternal characteristics (age, education, and parity), and household/concession characteristics (religion, ethnicity, distance to health center, and economic status ranking) were examined as possible explanatory variables in each of the models. Caregiver report of fever was used; children's temperatures were not measured.

## Results

### Characteristics of the Sample

**Figure 1:**
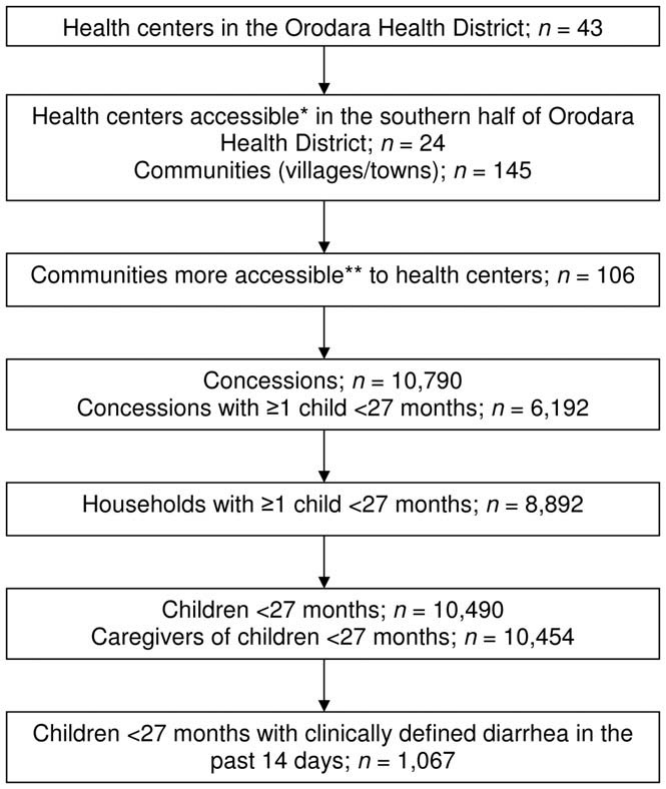
Summary of sampling framework for the survey in rural southwestern Burkina Faso. The sampling framework for the participation of health centers, communities, concessions, households, and children in the survey. Health centers and communities were first selected based on their accessibility by vehicle. In each community, concessions and households were visited to identify children <27 months of age and to interview their primary caregivers.

As summarized in Figure 1, the survey team visited a total of 10,790 concessions comprised of 14,287 households, of which 8,892 included at least one child <27 months. The total number of households visited represented 100% of those identified by local guides. A total of 10,454 caregivers of 10,490 children <27 months in these households were identified. Of the 10490 included in the survey, 198 children were excluded from the analyses of caregiver recognition of diarrhea because the caregiver didn't know the number of liquid/semi-liquid stools the child had on the day prior to the survey; 1,067 of the children had a caregiver-reported episode of clinically defined diarrhea in the preceding two weeks and were included in the analyses of care seeking and treatment practices. The demographic and economic characteristics of the children and their households are presented in Table 1 and Table S1. The vast majority of respondents (91.6%) were the child's mother, although other family members sometimes provided the requested information. Over half (54.3%) of the households surveyed were located in the same town as one of the public sector health centers.

**Table 1:** Demographic and economic characteristics of the sample.

	Total child sample (N = 10,490)		Children with clinically defined diarrhea episode in previous 2 weeks (N = 1,067)	
Characteristic	N	%	N	%
**Mother's age (years), ** ***n*** ** = 10,454 women**				
<20	1303	12.4	147	13.8
20–29	5353	51	559	52.4
30–39	2750	26.2	260	24.4
40–49	428	4.1	49	4.6
**Household religion, ** ***n*** ** = 8,892 households**				
Muslim	8273	78.9	850	79.7
Traditional	1448	13.8	145	13.6
Christian	742	7.1	71	6.7
**Mother's formal education completed, ** ***n*** ** = 10,454 women**				
None, non-literate	7498	71.5	724	67.9
None, literate	903	8.6	110	10.3
Primary	1551	14.8	173	16.2
Secondary or higher	520	5	60	5.6
**Distance of child's village of residence to health center (one way, km)**				
0 (living in same town, village)	5695	54.3	542	50.8
>0 and <5	1234	11.8	143	13.4
5–10	1583	15.1	163	15.3
>10	1978	18.9	219	20.5
**Child sex**				
Male	5234	49.9	578	54.2
Female	5204	49.6	487	45.6
**Child age (months)**				
<2	899	8.6	24	2.3
2–3	950	9.1	57	5.3
4–5	868	8.3	70	6.6
6–11	2310	22	308	28.9
12–17	2555	24.4	308	28.9
18–23	2019	19.3	212	19.9
24–27	792	7.6	81	7.6

Numbers may not sum due to missing values. Difference between indicated N and number of observations represents missing or unknown information.

### Prevalence and Caregiver Recognition of Diarrhea

Of the 10262 children included, 784 (7.6%) had clinically defined diarrhea according to the WHO criteria of ≥3 liquid or semi-liquid stools/day on the previous day. [3] By contrast, the caregivers reported independently that just 428 of these 784 children (54.6%) had diarrhea on the previous day, and they thought that diarrhea was present in 273 children (2.9% of 9478) who did not fulfill the WHO clinical criteria. The estimated prevalence of diarrhea was slightly less according to the caregivers' definition (701 of 10262; 6.8%) compared with the clinical definition (7.6%) (*P*<0.001, McNemar's test). More than one-fifth (21.6%) of caregivers of children with clinically defined diarrhea on the previous day reported multiple symptoms (i.e., diarrhea with ≥2 of the following illness symptoms: fever, vomiting, anorexia, bloody stool, or ≥6 liquid/semi-liquid stools in the previous 24 hours) during their child's illness episode.

**Figure 2:**
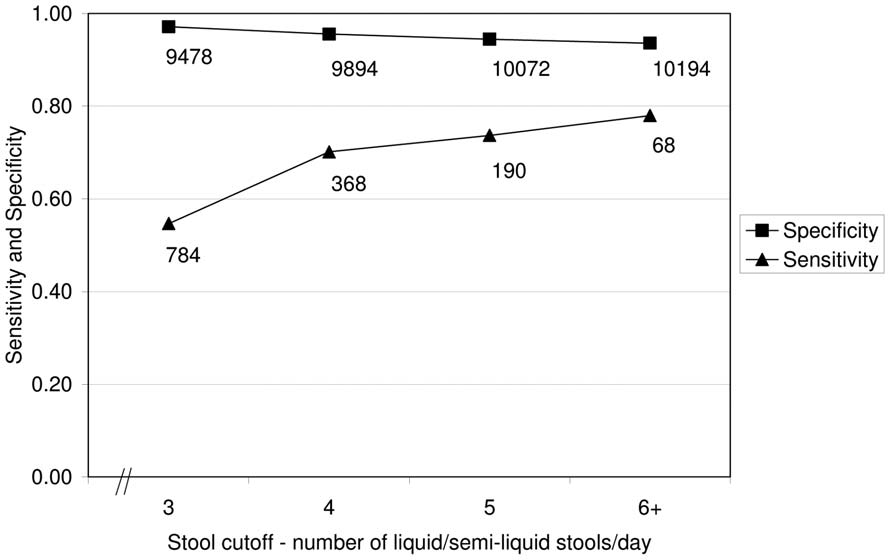
Sensitivity and specificity using different cutoffs for number of liquid/semi-liquid stools to define diarrhea. The sensitivity and specificity of caregiver diagnoses are shown for different cutoffs of reported frequency of liquid or semi-liquid stools. Caregiver diagnostic sensitivity increased from 0.55 using the cutoff of 3 stools per day to 0.78 when ≥6 stools were reported. Specificity was consistently >0.93.

Caregiver recognition of diarrhea increased if a higher number of liquid stools was used as the threshold to define illness. In particular, the sensitivity of caregivers' ability to recognize diarrhea increased from 0.55 using the cutoff of 3 stools per day to 0.78 when ≥6 stools were reported (Figure 2). Specificity was consistently >0.93. Caregivers recognized diarrhea more frequently when fever [adjusted odds ratio (OR) = 3.17, 95% confidence interval (CI) (2.29, 4.38)], vomiting [adjusted OR = 1.84, 95% CI (1.24, 2.73)], or anorexia [adjusted OR = 2.27, 95% CI (1.57, 3.30)] was also present. Sensitivity and specificity of the caregiver diagnoses also differed significantly by the child age. Whereas only 10.6% and 33.8% of cases with ≥3 liquid/semi-liquid stools were reported as diarrhea among children less than 2 months of age and 2–3 months of age, respectively, more than 50% of such cases were diagnosed correctly among children 4–27 months of age (*P*<0.001). In bivariate analyses, mothers with no formal education or only primary school attendance reported correctly that their children had diarrhea more frequently than mothers with secondary education or higher (56.6% and 53.8% vs. 34.0%, *P* = 0.008). Fewer women from households with traditional religious beliefs correctly recognized the presence of diarrhea (44.2%) than caregivers from Muslim (58.6%) or Christian households (55.4%) (*P* = 0.04). There was no significant difference in recognition of diarrhea by household economic status, distance to health center, or mother's age or parity. In the multiple logistic regression model, household ethnicity, mother's education, child age and fever were significant predictors of caregiver recognition of diarrhea (Table 2).

**Table 2:** Results of a multiple logistic regression analysis for factors predicting caregiver recognition of clinically defined diarrhea (N = 1,067).

Characteristic	OR* (95% CI)	*P*
**Fever**		
Present (*n* = 700)	2.72 (1.92, 3.87)	<0.0001
Absent (*n* = 367)	Ref.	
**Mother's formal education completed**		
None (*n* = 834)	2.74 (1.39, 5.41)	0.015
Primary (*n* = 173)	2.42 (1.11, 5.28)	
Secondary or higher (*n* = 60)	Ref.	
**Household ethnic group**		
Senoufo (*n* = 283)	0.89 (0.51, 1.57)	0.011
Mossi (*n* = 195)	1.61 (0.85, 3.03)	
Siamou (*n* = 142)	1.08 (0.57, 2.02)	
Toussiant (*n* = 105)	1.78 (0.84, 3.78)	
Samos (*n* = 113)	1.18 (0.59, 2.36)	
Dioula (*n* = 43)	0.72 (0.29, 1.76)	
Peulh (*n* = 52)	1.90 (0.79, 4.58)	
Bobo (*n* = 44)	5.11 (1.68, 15.54)	
Other (*n* = 90)	Ref.	
**Child's age (months)**		
<6 (*n* = 151)	0.27 (0.19, 0.39)	<0.0001
6–27 (*n* = 909)	Ref.	

* Each odds ratio is adjusted for each other characteristic included in the table. Independent variables included in the model were the presence of fever, vomiting, and decreased appetite, mother's education, household ethnic group, household religion and child sex. Clinically defined diarrhea = ≥3 liquid or semi-liquid stools/d. Ref. = reference category for each independent variable.

The misdiagnosis of caregiver-defined diarrhea in children who excreted fewer than three liquid or semi-liquid stools per day (“false positives”) was more common when other illness symptoms were present. The adjusted odds of misdiagnosis when fever [adjusted OR = 2.27, 95% (1.68, 3.08)], vomiting [adjusted OR = 2.56, 95% CI (1.79, 3.67)], or decreased appetite [adjusted OR = 2.80, 95% CI (1.99, 3.95)] was present were twice those when these symptoms were absent, and nearly eight-fold greater when blood was present in stool [adjusted OR = 7.71, 95% CI (4.01, 14.83)].

### Care seeking Behaviors

During the 14-day period prior the interview, 1067 of 10490 children (10.2%) reportedly had clinically defined diarrhea. Just over half (54.9%) of the caregivers of these children stated that they sought care outside the home, mostly by consulting care providers in public sector health clinics (77.5% of episodes), or visiting a traditional healer or a market/ambulatory drug vendor (22.0%). Among those who sought assistance outside the home, 72.7% did so within the first 72 hours of illness.

The most important factors associated with any care seeking outside the home were the reported presence of associated clinical symptoms (fever, vomiting, and anorexia), longer duration episodes, lower maternal parity, shorter distance to the public health center, and male child sex (Table 3). The odds of seeking assistance from a formal health care provider (public sector health center, hospital, or pharmacy) – as opposed to a traditional healer, a market vendor, or not seeking care outside the home – were higher if the child was reported to have fever [adjusted OR = 2.00, 95% CI (1.49, 2.69)], vomited [adjusted OR = 1.60, 95% CI (1.20, 2.12)], or a longer duration of illness [adjusted OR = 1.90, 95% CI (1.37, 2.64)] and when the family resided closer to the health center [adjusted OR = 1.81, 95% CI (1.28, 2.56)].

**Table 3:** Results of a multiple logistic regression analysis for factors predicting any care seeking outside the home for clinically defined childhood diarrhea (N = 1,067).

Characteristic	OR* (95% CI)	*P*
**Fever**		
Present (*n* = 700)	1.90 (1.42, 2.55)	<0.0001
Absent (*n* = 367)	Ref.	
**Vomiting**		
Present (*n* = 323)	1.53 (1.14, 2.06)	0.005
Absent (*n* = 740)	Ref.	
**Appetite lower than usual**		
Present (*n* = 617)	1.53 (1.16, 2.00)	0.002
Absent (*n* = 443)	Ref.	
**Duration of diarrhea episode**		
<3 days (*n* = 281)	Ref.	<0.001
3–7 days (*n* = 533)	1.62 (1.18, 2.22)	
8–14 days (*n* = 217)	2.03 (1.38, 2.99)	
≥15 days (*n* = 27)	4.21 (1.61, 11.04)	
**Mother's parity**		
1–2 (*n* = 445)	Ref.	0.003
3–4 (*n* = 320)	0.70 (0.51, 0.96)	
≥5 (*n* = 302)	0.58 (0.42, 0.80)	
**Distance of child's village of residence to health center (one way, km)**		
0 (same town or village; *n* = 542)	Ref.	0.010
>0 and <5 (*n* = 143)	0.90 (0.60, 1.34)	
5–10 (*n* = 163)	1.12 (0.77, 1.65)	
>10 (*n* = 219)	0.59 (0.42, 0.83)	
**Child sex**		
Male (*n* = 578)	1.33 (1.02, 1.73)	0.0336
Female (*n* = 487)	Ref.	

* Each odds ratio is adjusted for each other characteristic included in the table. Independent variables included in the model were the presence of fever, vomiting, and decreased appetite, duration of diarrhea episode, mother's parity, distance from child's residence to public health center, and child sex.

### Treatment

Among the 1067 children with clinically defined diarrhea during the past two weeks, 79.6% were reportedly given some form of treatment. Of those who received treatment, 59.0% received one form of treatment, 26.8% received two, and 14.3% received three or more (caregivers could provide up to three responses). Traditional therapies, including oral and topical medicines made from plants (leaves, stems, roots) and amulets placed around the waist or wrist, were among the most commonly administered treatments (29.5% of children). ORS was given to 24.4% of children; and other medications, including antibiotics and unidentified drugs (27.6% of children), were administered to 41.2% of children. ORS was provided more frequently to children with ≥6 stools/d (32.8%) than to those with less severe (i.e., 3–5 stools/d) and uncomplicated diarrhea (i.e., without reported fever, bloody stool, vomiting, or anorexia, 18.0%).

The specific forms of treatments that were offered depended on the type of health care provider consulted (Table 4). Health center personnel recommended ORS more frequently (46%) than other care providers, but they also prescribed antibiotics and other medicines more frequently. Traditional practitioners most frequently prescribed some form of traditional therapy; only ∼12% of children seen by a traditional practitioner were given ORS. Factors that were associated with reported ORS use were longer duration of the diarrheal episode [adjusted OR = 2.59 (95% CI: 1.08, 6.21)] for episodes lasting ≥15 days in duration compared to episodes <3 days in duration) and older child age [adjusted OR = 0.47 (95% CI: 0.29, 0.77)] for children <6 months of age vs. children 12–27 months of age). Of the caregivers who reported not seeking care outside the home, ∼8% gave ORS, 46% gave traditional therapies, and one-third reportedly did not provide any form of treatment.

**Table 4:** Percent of child caregivers who reported using different forms of treatment for clinically defined childhood diarrhea by type of care provider consulted (N = 1,067).

Treatment given*	Formal** provider, *n* = 455	Traditional practitioner, *n* = 69	Market vendor, *n* = 60	No care provider sought outside the home, *n* = 481	*P*
Traditional therapy	5.3	88.4	28.8	46	<0.0001
Oral rehydration solution	45.9	11.6	5	8.2	<0.0001
Anti-diarrheal	25.7	2.9	30.5	1.7	<0.0001
Antibiotic	28.1	1.5	10.2	1.9	<0.0001
Anti-malarial	19.3	0	11.9	1.9	<0.0001
Other medicine, type unidentified	51.9	14.5	30.5	14	<0.0001
None	0.9	1.5	3.7	35.3	<0.0001

* Up to three responses accepted; numbers may not sum to 100% due to missing values.

** Includes public health center or hospital, pharmacy, or private doctor.

The odds of receiving any treatment among children who resided in the same village as the health center were twice the odds for those residing in villages >10 kilometers (km) from the health center (Table 5). Children with the greatest odds of receiving traditional therapy were those who lived ≥5 km from the health center compared with those who lived closer (<5 km) [adjusted OR = 1.58, 95% CI (1.18, 2.11)].

**Table 5:** Results of a multiple logistic regression analysis for factors predicting any treatment given to child for clinically defined childhood diarrhea (N = 1,067).

Characteristic	OR* (95% CI)	*P*
**Number of other illness symptoms**		
0 (*n* = 189)	Ref.	<0.0001
1 (*n* = 268)	1.98 (1.25, 3.13)	
2 (*n* = 368)	2.99 (1.90, 4.71)	
≥3 (*n* = 228)	3.05 (1.80, 5.15)	
**Distance of child's village of residence to health center (one way, km)**		
0 (same town, village; *n* = 542)	Ref.	0.001
>0 and <5 (*n* = 143)	0.68 (0.41, 1.13)	
5–10 (*n* = 163)	1.09 (0.63, 1.89)	
>10 (*n* = 219)	0.47 (0.31, 0.70)	

* Each odds ratio is adjusted for each other characteristic included in the table. Independent variables included in the model were the presence of fever, vomiting, and decreased appetite.

The number of concurrent illness symptoms present was the strongest predictor of antibiotic use. The adjusted OR was 5.05 (95% CI: 2.31, 11.06) when diarrhea was accompanied by ≥3 other illness symptoms compared with uncomplicated diarrhea. Antibiotic use was not associated with the presence of reported dysentery, fever, or other specific symptoms when each of these factors were included in a logistic regression model as possible explanatory factors.

### Reported Knowledge of ORS

Approximately two thirds of the caregivers of children with recent diarrhea stated that they had heard of ORS. Caregivers with the greatest odds of reporting ORS awareness were those who lived closer to the health center [adjusted OR = 2.35, 95% CI (1.77, 3.12)], those from higher economic status households [adjusted OR = 1.53, 95% CI (1.09, 2.14)], and those with older (12–17 months old) children [adjusted OR = 1.64, 95% CI (1.10, 2.44)]. Among caregivers familiar with ORS, 83.7% reported that they were informed of ORS at the health center. Other frequently reported sources of information about ORS were radio or television (35.1% of caregivers) and another parent or friend (30.3% of caregivers). Among those who administered ORS, the predominant ORS suppliers were the pharmacy (54.7%) and the health center (39.5%).

### Feeding Practices

A total of 994 of the 1067 children (93.2%) with clinically defined diarrhea in the previous two weeks were breastfed at the time of the interview, and 98.8% of these children continued to breastfeed during the diarrhea episode. However, only 35.9% of caregivers reported giving the sick child more fluids than usual, and less than half (46.8% of caregivers) reported continued or increased feeding the child's usual diet during diarrhea. A greater proportion of children were given more fluids with longer illness duration [adjusted OR = 1.38, 95% CI (1.05, 1.83)] or when clinically defined diarrhea was accompanied by anorexia [adjusted OR = 2.52, 95% CI (1.41, 4.50)]. Children <6 months of age had only one-third the odds of receiving extra fluids as older children [adjusted OR = 0.38, 95% CI (0.24, 0.60)]. Fewer children were offered their usual quantity of food to eat during diarrhea in the presence of fever [adjusted OR = 0.74, 95% CI (0.56, 0.98)] and anorexia [adjusted OR = 0.37, 95% CI (0.28, 0.48)].

## Discussion

The results of this survey indicate that child caregivers in rural areas of southwestern Burkina Faso may fail to recognize nearly half the episodes of diarrhea that occur in their young children and only half of those who recognize illness seek care outside the home. While the majority of caregivers (80%) reportedly treat illness, treatment practices do not correspond with IMCI recommendations. The caregivers' ability to recognize episodes of clinically defined diarrhea, [3] varied by household, caregiver, child, and disease characteristics, as did external care seeking and home-treatment decisions. Caregivers recognized the presence of diarrhea more frequently when additional signs of severity, such as greater stool number, and the presence of fever, vomiting, and anorexia, accompanied the illness. Similarly, caregivers were more likely to report diarrhea among children with fever, vomiting, poor appetite, or fecal blood even when the reported stool pattern did not meet the clinical definition of diarrhea. Thus, the presence of these other illness symptoms seems to be important for fulfilling the local concept of diarrhea (*“konoboli”*).

The majority of caregivers who sought treatment outside the home used the local public sector health centers, but only 24.4% of children received ORS; therapeutic zinc supplementation was not yet available in the Orodara Health District. Even among the subset of caregivers with reported knowledge of ORS, only 41% of those who correctly recognized that the child had diarrhea on the previous day reportedly provided ORS. Given the availability of ORS in the public health sector, the reasons for the low ORS utilization are unclear and warrant further study. 14% of clinic attendees received an antibiotic even though specific signs of dysentery or systemic infection were not reported, suggesting that antibiotics may have been overprescribed in the clinic. Thus, health center staff may require retraining in general aspects of diarrhea treatment.

Major strengths of this survey are the large sample size and wide range of factors considered as possibly affecting caregiver recognition and treatment of childhood diarrhea. However, there are also several limitations to the study that must be recognized. First of all, the study sample was purposefully selected to include households that are relatively accessible to the health center, so that the children could be reached during the subsequent intervention trial. Thus, the sample is not representative of the entire local population, and the results may not apply to other parts of the country. Nevertheless, >85% of the people residing in the southern half of the Orodara Health District were covered by the survey, so the results should be generally representative of the local population, except for the most inaccessible households. Secondly, it is possible that the clinically defined cases of diarrhea were misclassified because the information on stool number and consistency and other associated symptoms depended on caregiver recall, and it was not possible to confirm the accuracy of these reports. Finally, the cross-sectional study design and two-week recall histories tend to over-represent longer duration episodes because caregivers are more likely to remember illnesses that are ongoing than ones that have resolved. [17] Caregiver failure to report previously resolved episodes of shorter duration could also affect the conclusions regarding case management practices if the shorter episodes were less likely to have been treated. Nevertheless, it is seems that the results are likely to provide an accurate overview of care seeking behaviors and treatment practices for those episodes that are treated.

The failure to recognize clinically defined diarrhea, particularly among caregivers of younger children is of concern because the incidence of dehydration and the case-fatality rate are greatest among younger children. [5] However, recognition of diarrhea improved when there was a greater number of liquid and semi-liquid stools, so those children at greatest risk of dehydration would be more likely to be diagnosed correctly. Likewise, children with associated symptoms that might require additional therapy, such as fever, vomiting, and anorexia, were more likely to be diagnosed correctly. However, our data suggest that even when children are sick with a combination of symptoms that would place them at high risk of dehydration (high stool frequency, fever, vomiting, or anorexia) appropriate treatment with ORS is utilized by less than one quarter of caregivers, suggesting that caregivers need training in recognizing symptoms requiring treatment.

Over half of the population in the study area reside close to a public sector health center and utilize these services, as was observed previously in Mali where ∼40% of caregivers visited a community health center or community health worker for treatment of childhood diarrhea. [18] These observations argue for a strategy focusing on public sector provision of diarrhea treatment services, including the planned introduction of therapeutic zinc supplements. However, families that reside further from the existing health centers tend to rely more on traditional practitioners or not seek treatment outside the home, so a separate approach might be necessary to reach these households. One such approach could be to train community health agents residing in each village, which would eliminate the obstacle of distance, but would require training and ongoing supervision of the health worker and monitoring and evaluation of the program. Another strategy could be to disseminate information directly to child caregivers through mass media channels, as over 70% of the households surveyed reported owning a radio or mobile telephone. Finally, the possibility of training traditional practitioners on recommended diarrhea treatment should be explored, as has been done recently in Bangladesh. [19]

In conclusion, interventions are needed both to enhance caregiver recognition of childhood diarrhea and to improve community-based and facility-based case management by the caregivers and local health practitioners. The best approaches to address these issues need further exploration and development.

## Supporting Information

Table S1 **Demographic and economic characteristics of the sample.**
(DOC)
