# Supplementary material for: Caregiver Recognition of Childhood Diarrhea, Care Seeking Behaviors and Home Treatment Practices in Rural Burkina Faso: A Cross-Sectional Survey
Source: PLoS One. 2012 Mar 13;7(3):e33273. doi: 10.1371/journal.pone.0033273 (PMC3302832; doi:10.1371/journal.pone.0033273)
Supplement: Table S1 — Demographic and economic characteristics of the sample. (DOC) [file pone.0033273.s001.doc]

| Table S1. Demographic and economic characteristics of the sample.   |  | | | | | |  | | --- | --- | --- | --- | --- | --- | --- | |  |  | |  |  | |  | |  | **Total child sample (N = 10,490)** | | | **Children with clinically defined diarrhea episode in previous 2 weeks (N = 1,067)** | | | | **Characteristic** | **N** | **%** | | **N** | **%** | | | **Household ethnic group, *n* = 8,892 households** |  |  | |  |  | | | Senoufo | 3260 | 31.1 | | 283 | 26.5 | | | Mossi | 1854 | 17.7 | | 195 | 18.3 | | | Siamou | 1226 | 11.7 | | 142 | 13.3 | | | Toussiant | 1124 | 10.7 | | 105 | 9.8 | | | Samos | 1007 | 9.6 | | 113 | 10.6 | | | Dioula | 428 | 4.1 | | 43 | 4 | | | Peulh | 420 | 4 | | 52 | 4.9 | | | Bobo | 310 | 3 | | 44 | 4.1 | | | Other | 848 | 8.1 | | 90 | 8.4 | | | **Mother's occupation, *n* = 10,454 women** |  |  | |  |  | | | Farmer and Housewife | 7020 | 66.9 | | 633 | 59.3 | | | Housewife | 1414 | 13.5 | | 166 | 15.6 | | | Merchant and Housewife | 1105 | 10.5 | | 137 | 12.8 | | | Farmer, Merchant, and Housewife | 578 | 5.5 | | 74 | 6.9 | | | Other | 373 | 3.6 | | 57 | 5.3 | | | **Mother's parity, *n* = 10,454 women** |  |  | |  |  | | | 1-2 | 4207 | 40.1 | | 445 | 41.7 | | | 3-4 | 3071 | 29.3 | | 320 | 30 | | | ≥5 | 3205 | 30.6 | | 302 | 28.3 | | | **Selected concession and household assets and infrastructure** |  |  | |  |  | | | Drinking water supply was tap, covered well or pump (wet season) | 5477 | 52.2 | | 525 | 49.2 | | | Sanitation facility was flush toilet or latrine | 6609 | 63 | | 689 | 64.6 | | | Floor material was cement or stone | 4408 | 42 | | 503 | 47.1 | | | Roof material was sheet metal/tin | 6544 | 62.4 | | 722 | 67.7 | | | Wall material was cement or stone | 1528 | 14.6 | | 160 | 15 | | | Principal source of lighting was electricity | 634 | 6 | | 82 | 7.7 | | | *Numbers may not sum due to missing values. Difference between indicated N and number of observations represents missing or unknown information. | | | | | | | |  |  |  |  |
| --- | --- | --- | --- | --- | --- | --- | --- | --- | --- | --- | --- | --- | --- | --- | --- | --- | --- | --- | --- | --- | --- | --- | --- | --- | --- | --- | --- | --- | --- | --- | --- | --- | --- | --- | --- | --- | --- | --- | --- | --- | --- | --- | --- | --- | --- | --- | --- | --- | --- | --- | --- | --- | --- | --- | --- | --- | --- | --- | --- | --- | --- | --- | --- | --- | --- | --- | --- | --- | --- | --- | --- | --- | --- | --- | --- | --- | --- | --- | --- | --- | --- | --- | --- | --- | --- | --- | --- | --- | --- | --- | --- | --- | --- | --- | --- | --- | --- | --- | --- | --- | --- | --- | --- | --- | --- | --- | --- | --- | --- | --- | --- | --- | --- | --- | --- | --- | --- | --- | --- | --- | --- | --- | --- | --- | --- | --- | --- | --- | --- | --- | --- | --- | --- | --- | --- | --- | --- | --- | --- | --- | --- | --- | --- | --- | --- | --- | --- | --- | --- | --- | --- | --- | --- | --- | --- | --- | --- | --- | --- | --- | --- | --- | --- | --- | --- | --- | --- | --- | --- | --- | --- | --- | --- | --- | --- | --- | --- | --- | --- | --- | --- | --- | --- | --- | --- | --- | --- | --- | --- | --- | --- | --- | --- | --- | --- | --- | --- | --- | --- | --- | --- | --- | --- | --- | --- | --- | --- | --- | --- | --- | --- | --- | --- | --- | --- | --- | --- | --- | --- | --- | --- | --- | --- | --- | --- | --- | --- | --- |
|  |  |  |  |  |
